# A Novel RNA Binding Protein-Related Prognostic Signature for Hepatocellular Carcinoma

**DOI:** 10.3389/fonc.2020.580513

**Published:** 2020-10-28

**Authors:** Yongbiao Huang, Sheng Chen, Wan Qin, Yali Wang, Long Li, Qianxia Li, Xianglin Yuan

**Affiliations:** ^1^ Department of Oncology, Tongji Hospital, Huazhong University of Science and Technology, Wuhan, China; ^2^ Department of Hepatobiliary Surgery, Affiliated Hospital of Hebei University, Baoding, China

**Keywords:** hepatocellular carcinoma, RNA binding proteins, prognostic signature, risk score, nomogram

## Abstract

Hepatocellular carcinoma (HCC) is a highly malignant and aggressive cancer with high recurrence rates and mortality. Some studies have illustrated that RNA binding proteins (RBPs) were involved in the carcinogenesis and development of multiple cancers, but the roles in HCC were still unclear. We downloaded the RNA-seq and corresponding clinical information of HCC from The Cancer Genome Atlas (TCGA) database, and 330 differentially expressed RBPs were identified between normal and HCC tissues. Through series of the univariate, the least absolute shrinkage selection operator (LASSO), and the stepwise multivariate Cox regression analyses, six prognosis-related key RBPs (CNOT6, UPF3B, MRPL54, ZC3H13, IFIT5, and PPARGC1A) were screened out from DE RBPs, and a six-RBP gene risk score signature was constructed in training set. Survival analysis indicated that HCC patients with high-risk scores had significantly worse overall survival than low-risk patients, and furthermore, the signature can be used as an independent prognostic indicator. The good accuracy of this prognostic signature was confirmed by the ROC curve analysis and was further validated in the International Cancer Genome Consortium (ICGC) HCC cohort. Besides, a nomogram based on six RBP genes was established and internally validated in the TCGA cohort. Gene set enrichment analysis demonstrated some cancer-related phenotypes were significantly gathered in the high-risk group. Overall, our study first identified an RBP-related six-gene prognostic signature, which could serve as a promising prognostic biomarker and provide some potential therapeutic targets for HCC.

## Introduction

Liver cancer, the fifth most frequent type of malignancy with high morbidity and mortality, has become the second leading cause of cancer death globally. It was estimated that 841,080 new liver cancer cases were diagnosed worldwide and 781,631 deaths occurred in 2018 (1, 2). Hepatocellular carcinoma (HCC), the main pathological type of primary liver cancer, represents approximately 80–90% of all liver cancer cases (3, 4). At present, the major treatments include systemic pharmacological treatment, surgical resection, transplantation, ablation therapies, transcatheter arterial chemoembolization, and radiotherapy (3, 5). In recent decades, the incidence and mortality of HCC has been increasing globally (1). In spite of the significant progress made in diagnosis and treatment, the prognosis for patients with HCC still remains poor due to the high complexity and heterogeneity of hepatocarcinogenesis (3). Therefore, it is critical to identify prognostic biomarkers and develop novel accurate prediction models for predicting prognosis of patients with HCC and guiding clinical therapy.

RNA binding proteins (RBPs) play a crucial role in post‐transcriptional gene regulation (6). RBPs can bind various types of RNAs include coding RNAs (mRNAs) and no-coding RNAs (rRNAs, ncRNAs, snRNAs, miRNAs, tRNAs, snoRNAs) through an RNA-binding domain directly (7, 8). So far, more than 1,500 human RBPs (7.5% of the proteome) have been identified that contain 600 structurally distinct RNA-binding domains (7). They form ribonucleoprotein complexes by binding their target RNAs and regulate RNA metabolism, include RNA maturation, splicing, transport, localization, polyadenylation, stability, degradation, and translation (8, 9). Most RBPs are evolutionarily conserved and ubiquitously expressed to maintain cellular homeostasis (7, 10). Due to the extremely significant biological function of RBPs, its dysfunction can lead to the occurrence of multiple diseases, including cardiovascular system diseases (11), blood diseases (10), neurodegenerative disorders (12), and cancers (11–14).

Previous published studies have indicated that aberrant expression of some RBPs can affect cell growth and proliferation and promote tumor occurrence and progression (15). In addition, its aberrant expression is also significantly related to malignant degree and clinical prognosis of patients with cancer (16). For instance, the RNA binding proteins Musashi-1 and Musashi-2 were found to be overexpressed in colorectal cancer, and they regulate the mRNA stability and translation in essential oncogenic signaling pathways (17). Negative elongation factor E (NELFE) promotes metastasis of pancreatic cancer through activating the Wnt/β-catenin signaling pathway and decreasing the NDRG2 mRNA stabilization (18). Human ribosomal protein S3 (RPS3) is upregulated in HCC and is closely relevant to the prognosis of patients with HCC. RPS3 stabilized SIRT1 mRNA through binding with the 3′ UTR of SIRT1 mRNA to sustaining HCC progression and the somatic copy-number alterations of NELFE enhanced MYC signaling and promote cell proliferation in HCC (19, 20). The molecular mechanism by which RBPs promote carcinogenesis and development is still not clear.

Consequently, we considered that RBPs were potential prognostic biomarkers for HCC patients. In our study, the RNA-seq data and corresponding clinical information of HCC cases were obtained from The Cancer Genome Atlas (TCGA) database, and then we identified differentially expressed RBPs between tumor and normal liver tissue. Based on differentially expressed RBPs, survival related RBPs were screened out and an RBP-associated prognostic model was constructed to predict the clinical outcome of HCC patients. The prognostic value of this model was validated in another HCC cohort from the International Cancer Genome Consortium (ICGC) database.

## Materials and Methods

### TCGA HCC Dataset and Difference Analysis 

The normalized RNA-seq data (Fragments Per Kilobase Million, FPKM) and corresponding clinical data, which contained 374 HCC samples and 50 normal liver tissue samples, were downloaded from TCGA database as training set. Wilcox Test was utilized to perform difference analysis and identify the differentially expressed RBPs (DE RBPs) between the HCC and normal tissue. RBPs with |log2 fold change (FC)| ≥ 0.5 and adj P-value < 0.05 were used for subsequent analysis.

### GO and KEGG Functional Enrichment Analyses

To explore main biological functions and signaling pathways of the differently expressed RBPs, the R package “clusterProfiler” was used to carry out Kyoto Encyclopedia of Genes and Genomes (KEGG) pathway and Gene Ontology (GO) enrichment analyses (21), and the results were visualized via “GOplot” R package. The false discovery rate (FDR) < 0.05 was thought to be statistically significant.

### PPI Network 

Protein-protein interaction (PPI) networks in differently expressed RBPs were constructed by using the STRING database and visualized via Cytoscape software (22). The Cytoscape plugin Molecular Complex Detection (MCODE) was used to detect the important modules in PPI network (23), and GO and KEGG analyses were conducted to further investigate their molecular function in HCC.

### Prognosis-Related Key RBPs Screening

The univariate Cox regression analysis was carried out to find the prognosis-related RBPs among the differentially expressed RBPs via “survival” R package, and P-value < 0.01 were considered for subsequent analysis, using the least absolute shrinkage selection operator (LASSO) regression analysis to further screen prognostic-related RBP genes with “glmnet” R package. Finally, the stepwise multivariate regression analysis was performed to screen out optimal key prognostic-related RBP genes and obtain their standardized regression coefficients.

### Survival, Expression, and Genetic Alteration Analyses of Key Prognosis-Related RBP Genes

The Kaplan–Meier curves survival was utilized to evaluate the prognostic value of each key RBP gene in TCGA cohort, and P-value < 0.05 was considered to have statistical difference. The copy-number alterations and mutations were detected with the online database cBioPortal (24), and the protein expression was detected by Human Protein Atlas (HPA) database (25).

### Construction of an RBP-Gene Prognostic Signature

A risk score signature was constructed by using multivariate Cox regression based on the previously obtained RBPs using the survival R package in TCGA. The risk score was calculated by the following formula: Risk score = Expression of gene1 × Coefficient of gene1 + Expression of gene2 × Coefficient of gene2 + … Expression of geneN × Coefficient of geneN (26, 27). By the median value of the risk score, all HCC patients were assigned into low-risk groups and high-risk groups, and the Kaplan-Meier curve analysis and log-rank test were used to assess the survival difference between two subgroups by “Survival” R package. The receiver operating characteristic (ROC) curves were plotted and the area under the curve (AUC) values were calculated with “SurvivalROC” R package, which was used to evaluate the predictive power (28). Then, the LIRI-JP project in ICGC dataset contained 229 HCC patient cases with complete clinical information and follow-time more than 1 month was used as testing set to validate the predictive capacity of this model (29). In addition, the univariate and multivariate Cox regression analyses were utilized to determine the correlation between RBP signature and clinical characteristics and OS in the TCGA and ICGC cohort, respectively. The statistical difference of risk scores between the stratified clinicopathologic features was calculated by using the Kruskal–Wallis test.

After that, a prognostic nomogram based on key prognosis-related RBP genes was generated by using “rms” R package to predict OS of HCC patients at 1-, 3-, and 5-years in the TCGA cohort. Meanwhile, the calibration curves were plotted to appraise the prognostic performance of the nomogram.

### Gene Set Enrichment Analysis

Gene set enrichment analysis (GSEA) was conducted with GSEA v4.0.3 software to identify different signaling pathways between two subgroups. Hallmark gene sets (h.all.v6.0.symbol.gmt) were downloaded from Molecular Signatures Database as the reference gene set (30). Nominal p-value < 0.05 and FDR q-value < 0.05 were set as the cut-off.

## Results

### Identification of the Differentially Expressed RBPs

**Figure 1:**
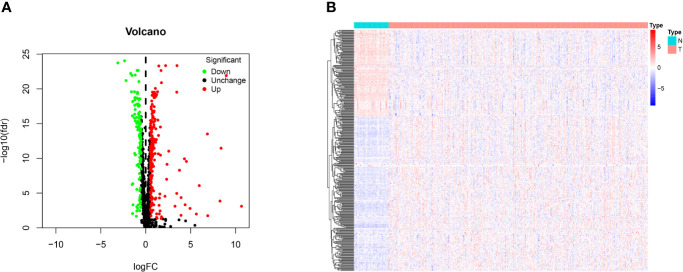
Identification of differentially expressed RNA binding proteins (DE RBPs) in TCGA dataset and enrichment analysis. **(A)** Volcano plot of all DE RBPs between HCC and normal samples, 208 were up-regulated and 122 were down-regulated. Red: up-regulated RBPs; Black: unchanged RBPs; Green: down-regulated RBPs. **(B)** Heat map of the DE RBPs based on their expression data log2 transformed FPKM values. The red represents high expression, and the green represents low expression.

The 1,542 human RBPs found so far were included in our study (7), a total of 330 DE RBPs were identified by Wilcox Test between 374 HCC tissues and 50 normal liver tissues, including 208 upregulated and 122 downregulated RBPs, according to the adj P-value < 0.05, |log2FC| ≥ 0.5 (**Figure 1**).

### Enrichment Analysis of DE RBPs

**Figure 2:**
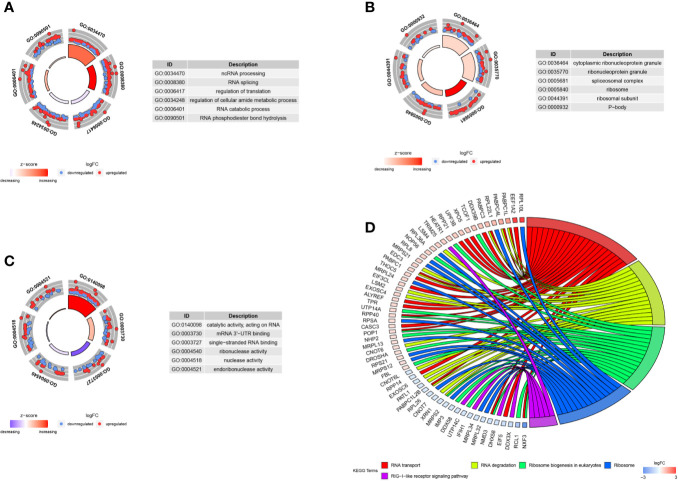
GO and KEGG enrichment analyses of DE RBPs. **(A–C)** Top six enriched GO terms respectively enriched in Biological processes (BP), Cellular components (CC), and Molecular functions (MF); **(D)** Five significantly enriched KEGG pathways for DE RBPs. The outer circle shows a scatter plot for each term or pathway of the logFC of the assigned genes, red circles represent up-regulation and blue represent down-regulation.

We carried out the GO and KEGG pathway enrichment analyses of the DE RBPs in HCC by using the R package “clusterProfiler.” GO analysis consists of biological process (BP), cellular component (CC), and molecular function (MF). The DE RBPs were significantly gathered in ncRNA processing, RNA splicing, regulation of translation, RNA catabolic process, and RNA phosphodiester bond hydrolysis of the BP category (**Figure 2A**); cytoplasmic ribonucleoprotein granule, ribonucleoprotein granule, spliceosomal complex, ribosome, ribosomal subunit, and P-body of the CC analysis (**Figure 2B**); RNA catalytic activity, mRNA 3'-UTR binding, single-stranded RNA binding, ribonuclease activity, nuclease activity, and endoribonuclease activity of the MF analysis (**Figure 2C**). The KEGG analysis results indicated that the DE RBPs were significantly gathered in RNA transport and degradation, mRNA surveillance pathway, spliceosome, ribosome, ribosome biogenesis in eukaryotes, and RIG-I-like receptor signaling pathway (**Figure 2D**). Many emerging studies have suggested that RBPs participate in RNA metabolism and formation of mRNA spliceosomal complex, and mediate post‐transcriptional gene regulation. The ribosome is a kind of ribonucleoprotein granule and is considered as a molecular machine for protein synthesis. Some RBPs are closely related to ribonucleoprotein formation, they can assemble specific RNAs to form ribonucleoprotein granules in eukaryotic cells, like P-bodies and stress granules. P-bodies are conserved cytoplasmic ribonucleoprotein granules in eukaryotic organisms, which involved in translational repression and mRNA decay and degradation (31–34). These results suggested that RBPs play an essential role in RNA processing and protein synthesis, and their aberrant expression could promote carcinogenesis and progression of a variety of tumors.

### PPI Network Analysis

**Figure 3:**
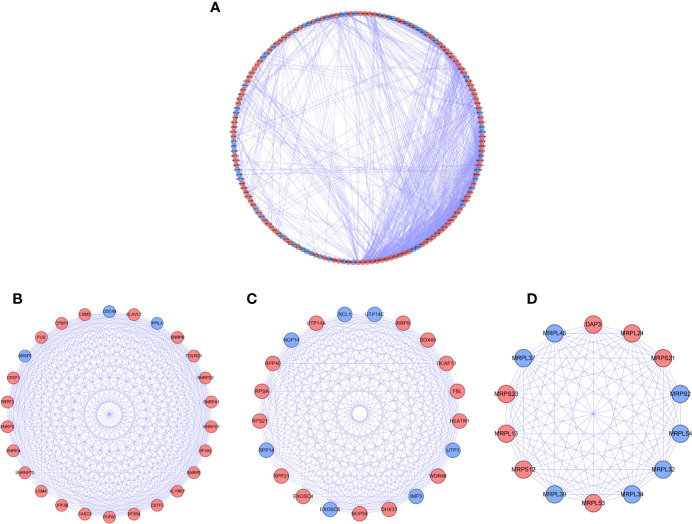
PPI network and modules analysis. **(A)** PPI network for DE RPBs; **(B)** Key module 1 in PPI network; **(C)** Key module 2 in PPI network; **(D)** Key module 1 in PPI network. Red: up-regulation, Blue: down-regulation.

For further understanding the function of DE RBPs in HCC procession, we constructed a PPI network that consists of 163 nodes and 1,047 edges by using STRING database and Cytoscape software (**Figure 3A**). Moreover, pivotal modules were identified from the PPI network using MODE plug in Cytoscape. Module 1 included 23 upregulated DE RBPs and 3 downregulated DE RBPs (**Figure 3B**), and enrichment analysis indicated they were correlated with RNA splicing, RNA 3'-end processing, and mRNA surveillance. Module 2 included 14 upregulated DE RBPs and 7 downregulated DE RBPs (**Figure 3C**), significantly enriched in ncRNA processing, rRNA processing, and ribosome biogenesis. Module 3 included 7 upregulated DE RBPs and 7 downregulated DE RBPs (**Figure 3D**), related to mitochondrial gene expression, mitochondrial translational termination, and elongation.

### Selection of Prognosis-Related RBPs

**Figure 4:**
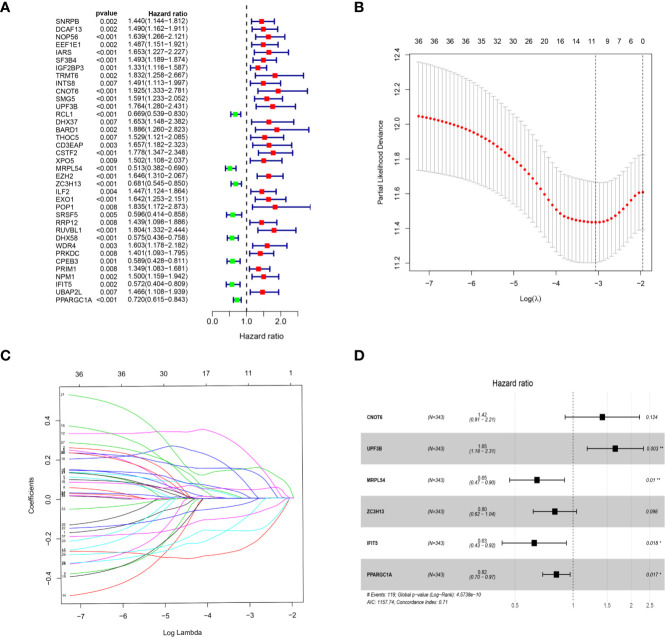
Selection of prognosis-related RBPs in the training cohort. **(A)** Univariate Cox regression analysis; **(B, C)** LASSO regression analysis; **(D)** Multivariate Cox regression analysis to screen out the key RBPs most relevant to prognosis.

We download the RNA-seq data and corresponding clinical information of HCC patients from the TCGA and ICGC databases, the TCGA HCC cohort as training set, the ICGC HCC cohort as testing set, and the clinical detailed characteristics were collated in **Table 1**. A total of 343 HCC cases with follow-up time more than 30 days in training set were included in the next series of analyses. The univariate Cox regression analysis was utilized to screen prognosis-related RBPs on DE RBPs by survival R package, and 37 survival-related RBPs among the DE RBPs were identified (p<0.01) (**Figure 4A**). Thereafter, the LASSO regression analysis was conducted for further decreasing the number of survival-related RBPs using 10-fold cross validation via “glmnet” R package (**Figures 4B, C**). Finally, we obtained six key prognosis-related RBP genes: CNOT6, UPF3B, MRPL54, ZC3H13, IFIT5, and PPARGC1A by stepwise multivariate regression analysis (**Figure 4D** and **Table 1**).

**Table 1:** The clinical Characteristics of HCC patients from TCGA and ICGC database.

Characteristics		Detailed data	
		TCGA cohort (n=343)	ICGC cohort (n=229)
**Status**			
	Dead	117 (34.11%)	40 (17.47%)
	Survival	226 (65.89%)	189 (82.53%)
**Age at diagnosis (years)**			
	≤65	219 (63.85%)	89 (38.86%)
	>65	124 (36.15%)	140 (61.14%)
**Gender**			
	Female	107 (31.20%)	61 (26.64%)
	Male	236 (68.80%)	168 (73.36%)
**Histological grade**			
	G1	53 (15.45%)	NA
	G2	164 (47.81%)	NA
	G3	113 (32.94%)	NA
	G4	13 (3.79%)	NA
**TNM stage**			
	I	164 (47.81%)	36 (15.72%)
	II	77 (22.45%)	106 (46.29%)
	III	81 (23.62%)	68 (29.69%)
	IV	3 (0.87%)	19 (8.31%)
	NA	18 (5.25%)	0 (0.00%)

NA, not available.

### Expression, Alteration and Survival Analyses of the Six Prognosis-Related RBP Genes

**Figure 5:**
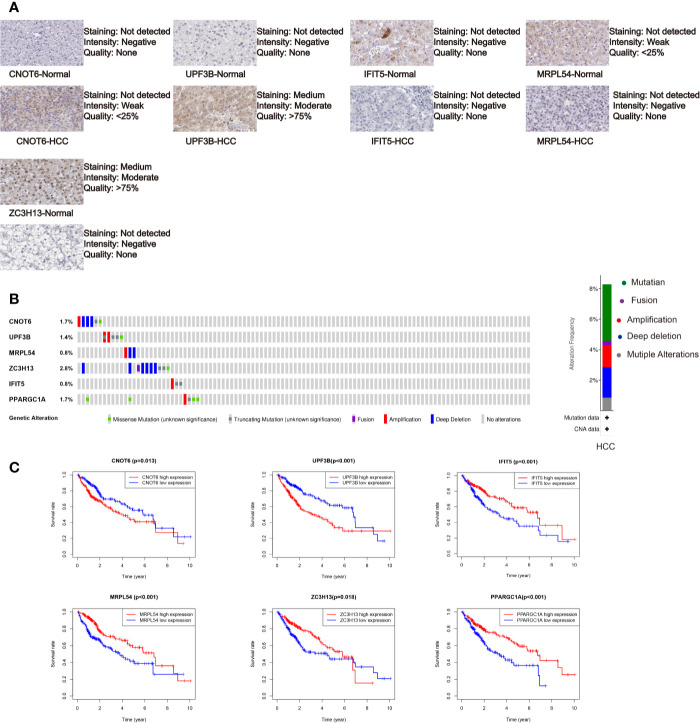
Comprehensive analysis of the six selected RBPs (CNOT6, UPF3B, MRPL54, ZC3H13, IFIT5, and PPARGC1A). **(A)** Immunohistochemistry of five RBPs using HPA database, except PPARGC1A. **(B)** Alteration analysis of these RBP genes. **(C)** Kaplan-Meier survival curves for the six genes.

We further analyzed the expression of these RBPs via HPA database, and the immunohistochemistry results of five key RBPs in HCC and normal tissues were presented in **Figure 5A**, with PPARGC1A not included in the database. By using the cBioPortal online database, we found that 39 out of the 366 HCC patients (11%) have genetic alterations (mutations and copy-number alterations) in the six RBP genes, and ZC3H13 with the highest alteration frequency (**Figure 5B**). The six key RBP genes were analyzed by using Kaplan–Meier curve analysis to further verify their prognostic value on the TCGA cohort, and the results demonstrated that HCC patients with UPF3B and CNOT6 low-expressions had longer OS, while patients with IFIT5, MRPL54, PPARGC1A, and ZC3H13 high-expression had better survival rate (**Figure 5C**).

### Construction Validation of the RBP-Related Risk Score Signature

Based on the previously obtained six key prognosis-related RBP genes, we established a risk core model, and the risk score of each HCC patients was calculated using the formula: Risk score = (0.34900 × CNOT6 Exp) + (0.50277 × UPF3B Exp) + (-0.43143 × MRPL54 Exp) + ( -0.21809 × ZC3H13 Exp) + ( -0.46413 × IFIT5 Exp) + ( -0.19919 × PPARGC1A Exp). Among these six prognosis-related RBPs, CNOT6 and UPF3B were high-risk factors (HR>1); MRPL54, ZC3H13, IFIT5, and PPARGC1A were protective factors (HR<1) (**Table 2**).

**Table 2:** The six prognosis-associated key RBPs identified by multivariate Cox regression analysis.

RBP name	Coefficient	HR	Lower 95% CI	Upper 95% CI	P-value
CNOT6	0.349007	1.417659	0.908826	2.21138	0.123921
UPF3B	0.502772	1.653298	1.18189	2.312733	0.003327
MRPL54	-0.431433	0.649577	0.468813	0.90004	0.009517
ZC3H13	-0.218092	0.804051	0.62116	1.040793	0.097658
IFIT5	-0.464131	0.628681	0.428497	0.922387	0.017643
PPARGC1A	-0.199195	0.81939	0.695361	0.965543	0.017375

HR, hazard ratio; CI, confidence interval.

**Figure 6:**
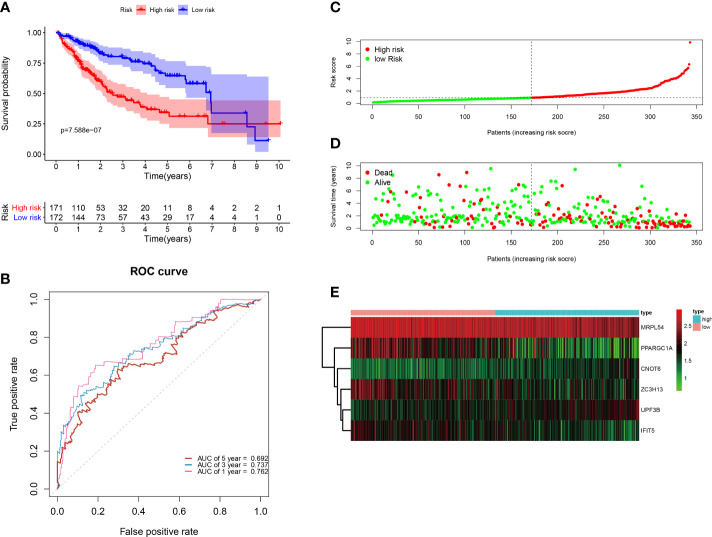
Construction of the six-RBP gene prognostic signature in the TCGA cohort. **(A)** Kaplan-Meier survival curve of HCC patients in the high- and low-risk groups. **(B)** ROC curves for predicting 1-, 3-, 5-year overall survival. **(C–E)** Distribution of risk score, survival time, and heat map of six genes expression.

All of the 343 HCC patients were assigned into high-risk (n = 171) and low-risk groups (n = 172) using the median risk score in the testing cohort. Low-risk patients had a significantly longer OS compared with the patients in high-risk group (p=7.588e−07) (**Figure 6A**). The AUC value for this six-RBP gene risk score signature was 0.762 in the 1-year ROC curve, 0.737 in the 3-year ROC curve, and 0.692 in the 5-year ROC curve (**Figure 6B**). The risk scores and survival status distribution of HCC patients between two subgroups were presented in **Figures 6C, D**. We found that as the risk score increased, the number of HCC deaths also increased in the training set. The heatmap of six RBP genes expression level was shown in **Figure 6E**.

**Figure 7:**
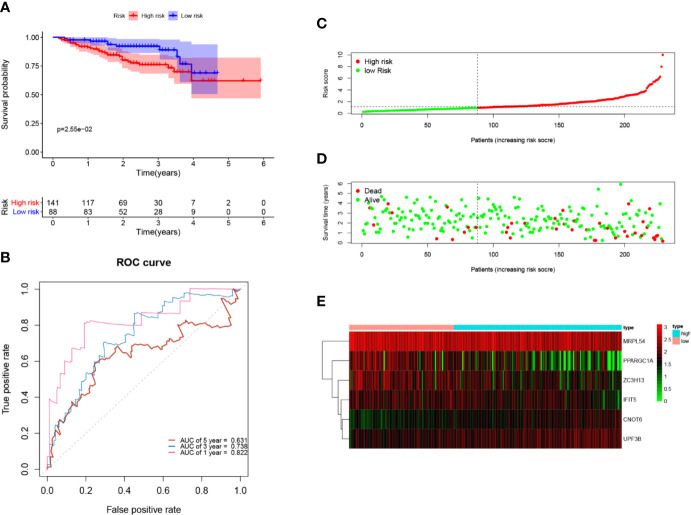
Validation of the six-RBP gene prognostic signature in the ICGC cohort. **(A)** Kaplan-Meier survival curve of HCC patients in the high- and low-risk groups. **(B)** ROC curves for predicting 1-, 3-, 5-year overall survival. **(C, E)** Distribution of risk score, survival time, and heat map of six gene expression.

Next, to further verify the prognostic performance of this model, we collected 229 HCC cases with follow-up time >30d as the testing set from the ICGC database, and we used the same formula to calculate their risk score. Same as TCGA cohort, according to the cut-off value of TCGA cohort, the results showed that patients with high-risk scores (n=141) had a worse OS than those in low-risk group (n=88) (p=2.55e−2), the AUC value of 1-, 3-, 5-year was 0.822, 0.738 and 0.631, respectively (**Figure 7**). These results indicated that our prognostic signature had considerable robustness in predicting OS for HCC patients.

### Association Between Clinical Characteristics and the Six-RBP Gene Signature 

**Figure 8:**
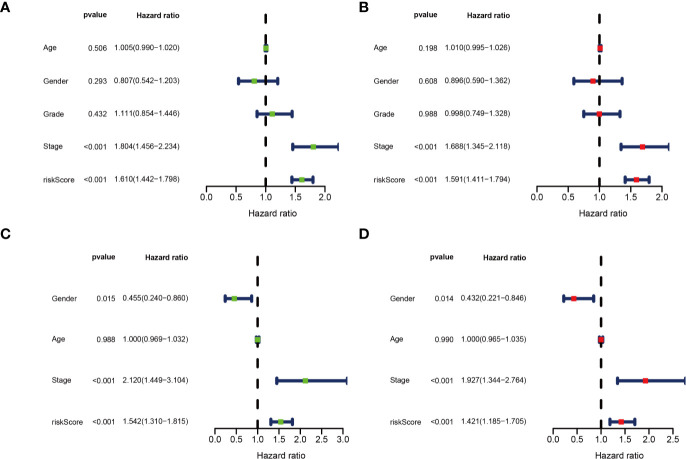
Identification of independent prognostic indicators. **(A)** Forest plots for Univariate Cox regression analysis; **(B)** Multivariate Cox regression analysis in TCGA cohort; **(C)** Forest plots for Univariate Cox regression analysis; **(D)** Multivariate Cox regression analysis in ICGC cohort.

Univariate and multivariate Cox regression analyses were performed for clinical features: age, gender, grade, stage, and risk score in training and testing set respectively. The results demonstrated the stage (P<0.001) and risk score (P<0.001) were independent prognostic indicators in the TCGA cohort (**Figures 8A, B** and **Table 3**), whereas in the ICGC cohort, the gender (P=0.014352), stage (P<0.001), and risk score (P<0.001) were independent prognostic indicators (**Figures 8C, D** and **Table 3**).

**Table 3:** Univariate and multivariate analyses of different clinical characteristics in TCGA and ICGC cohorts.

TCGA cohort	Univariate Cox analysis			Multivariate Cox regression		
	HR	95%CI	P-value	HR	95%CI	P-value
Age	1.005	0.990–1.020	0.50645	1.01	0.994–1.026	0.198323
Gender	0.807	0.542–1.203	0.293326	0.896	0.590–1.361	0.608359
Histological grade	1.111	0.854–1.446	0.43213	0.998	0.749–1.328	0.988103
TNM stage	1.804	1.456–2.234	<0.001	1.687	1.345–2.117	<0.001
Risk score	1.61	1.442–1.798	<0.001	1.591	1.411–1.794	<0.001
**ICGC cohort**	**Univariate Cox analysis**			**Multivariate Cox regression**		
	**HR**	**95%CI**	**P-value**	**HR**	**95%CI**	**P-value**
Age	1	0.969–1.032	0.987595	1	0.965–1.035	0.990104
Gender	0.454	0.240–0.860	0.015353	0.432	0.221–0.846	0.014352
Histological grade	NA	NA	NA	NA	NA	NA
TNM stage	2.12	1.448–3.104	<0.001	1.927	1.344–2.764	<0.001
Risk score	1.542	1.310–1.815	<0.001	1.421	1.185–1.705	<0.001

HR, hazard ratio; CI, confidence interval; NA, not available.

**Figure 9:**
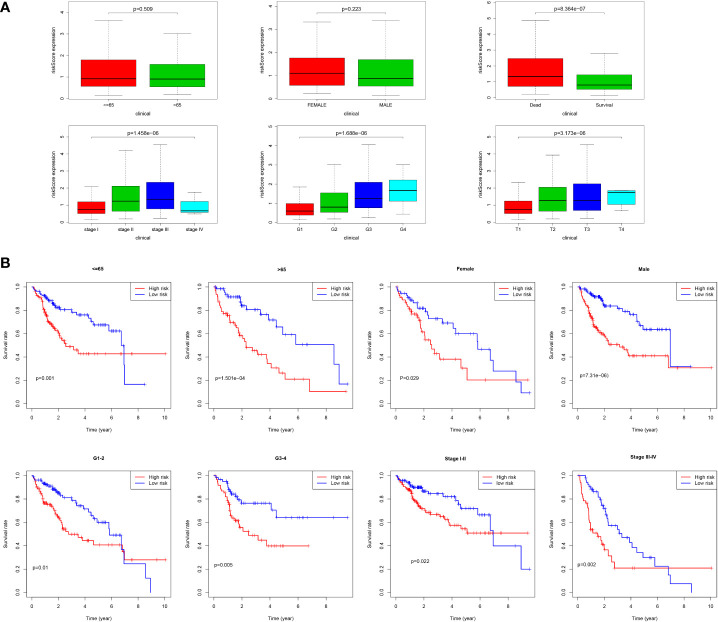
Correlation of risk score and clinical characteristics. **(A)** Risk score distribution between different clinical characteristics; **(B)** Kaplan-Meier survival analysis of the signature stratified by clinical characteristics.

As shown in **Figure 9A**, we found that most dead patients had higher risk scores, which suggested that high-risk patients usually had worse clinical outcomes. Moreover, HCC patients with advanced tumor clinicopathological parameters (stage II and stage III, G3 and G4, pT3 and pT4) were more likely to have higher risk scores than patients with early-stage HCC. We conducted further survival analyses that were stratified by clinical characteristics, and patients in the low-risk group had greater OS than high-risk in all clinical characteristics for stratification survival analyses, including age, gender, grade, and stage (**Figure 9B**).

### A Nomogram Establishment on the Six Key Prognosis-Related RBP Genes

**Figure 10:**
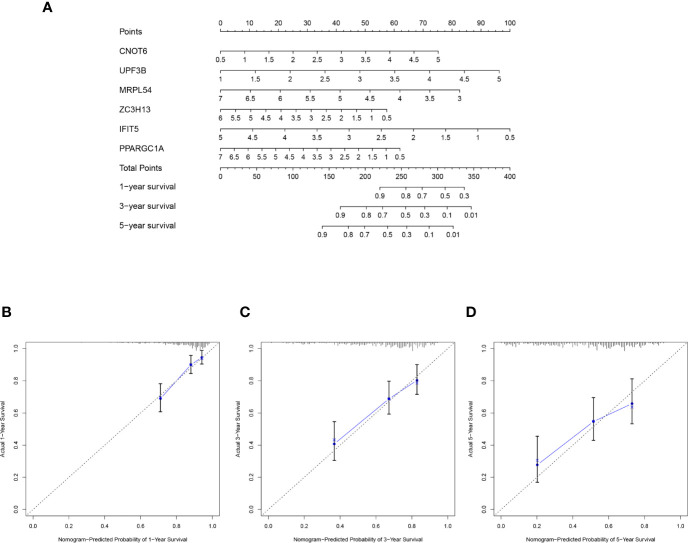
A nomogram in TCGA HCC dataset. **(A)** The nomogram was built based on this six-RBP gene signature in the training cohort. **(B–D)** The calibration plots showed good predictive performance for OS at 1-, 3-, 5-year.

The selected six key prognosis-related RBP genes were used to establish a prognostic nomogram through the multivariate Cox regression analysis. We can plot a perpendicular line between the total points axis and each prognostic axis, and estimated the survival probability of HCC patients at 1-, 3-, and 5-year (**Figure 10A**). We also drew the calibration curves, which indicated that nomogram had good prediction performance in HCC patients (**Figure 10B**).

### GSEA Analysis

**Figure 11:**
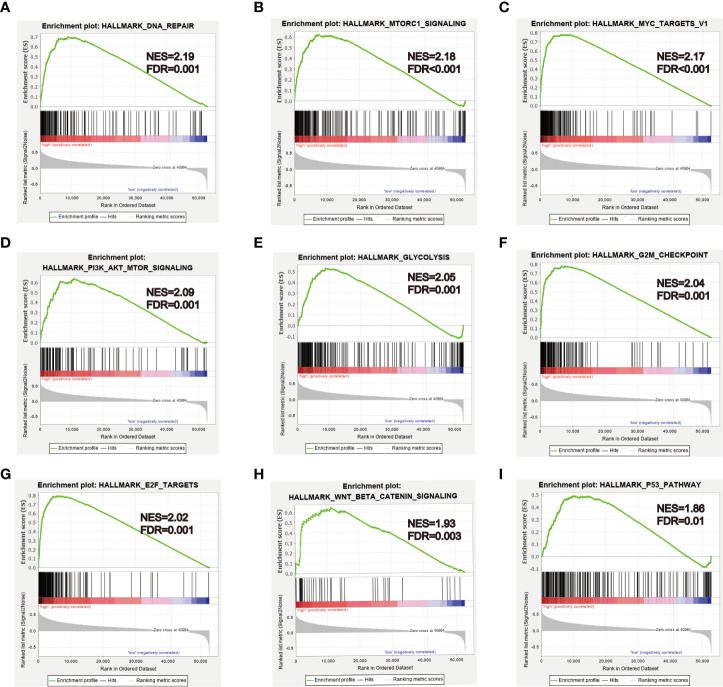
GSEA analysis between high- and low-risk groups. **(A–I)** Some cancer-related pathways were gathered in high-risk group: “DNA REPAIR,” “MTORC1_SIGNALING,” “MYC_TARGETS_V1,” “PI3K_AKT_MTOR SIGNALING,” “GLYCOLYSIS,” “G2M_CHECKPOINT,” “E2F_TARGETS,” “WNT_BETA_CATENIN_SIGNALING,” “P53_PATHWAY.” NES, Normalized enrichment score.

To further explore biological functions and pathways correlated with the risk score signature, GSEA was carried out between high- and low-risk groups in the TCGA HCC cohort. Some cancer-related gene sets were significantly gathered in HCC patients with high risk score, including “DNA repair,” “MYC targets V1,” “mTORC1 signaling,” “PI3K-AKT-mTOR signaling,” “glycolysis,” “G2M checkpoint,” “E2F targets,” “Wnt/beta-catenin Signaling,” “P53 pathway,” shown in **Figure 11**.

## Discussion

HCC has become a severe health concern in China; its incidence and mortality rate are still gradually rising due to hepatitis virus infection (1–3). Some research has shown that RBPs were closely related to the tumorigenesis and development of multiple cancers, but what role RBPs play in HCC were still unclear (11–14). In this study, we integrated the RNA-seq data of HCC from TCGA database, then identified 330 DE RBPs. The PPI network and functional enrichment analysis were conducted to explore the biological function and signaling pathways of DE RBPs in HCC. Next, we filtered out six key RBP genes (CNOT6, UPF3B, MRPL54, ZC3H13, IFIT5, and PPARGC1A) that were most relevant to prognosis by using the univariate, LASSO, and multivariate Cox regression analyses. Based on the six prognosis-related RBP genes, we established a promising six-RBP gene signature and nomogram to predict OS of HCC patients and validated its robustness in the ICGC cohort. The HCC patients were assigned into two subgroups, high- and low-risk groups, and patients in the high-risk group had poorer outcomes. Next, the GSEA analysis was utilized to investigate the differences in some critical signaling pathways between two subgroups in the TCGA HCC cohort.

Among the six key RBP genes, CNOT6 and UPF3B were highly expressed in the HCC tissues relative to the normal tissues and were considered as unfavorable factors that may lead to worse overall survival. Four genes (MRPL54, ZC3H13, IFIT5, and PPARGC1A) were downregulated and may function as tumor suppressor genes in HCC, and showed a positive correlation with prognosis. CNOT6 encodes Ccr4a protein that was a deadenylase subunit of the CCR4-Not complex (5, 35), and the CNOT6 rs2453176 C>T polymorphism was related to an increased risk of lung cancer (36). Previous research found that CNOT6 was overexpressed in non-metastatic lung squamous cell carcinoma, and it may be associated with low invasiveness (37). Moreover, the CNOT6 expression level was significantly lower in acute leukemia patients than healthy controls (38). UPF3B encodes a protein that participated in nonsense-mediated mRNA decay, and the mutation of UPF3B was associated with mental retardation (39). However, the role of UPF3b in cancer has not yet been reported and needs further study. López et al. used the machine-learning classification model to recognize that MRPL54 may be strongly connected to breast cancer (40). In our study, we found for the first time that UPF3b and MRPL54 were independent prognostic indicators in HCC. Liu et al. suggested ZC3H13 was downregulated in HCC, and patients with lower ZC3H13 expression had poorer overall survival, consisten with our findings (41). ZC3H13 also has been demonstrated to have prognostic value in other cancer types, such as lung adenocarcinoma, clear cell renal carcinoma, and anal squamous cell carcinoma (42–44). ZC3H13 expression was higher in lung adenocarcinoma, and its expression pattern was the same as that in HCC. However, ZC3H13 had a lower expression level in clear cell renal carcinoma. Trilla et al. suggested that the genetic variant of ZC3H13 was associated with poor disease-free survival. IFIT5 belongs to the interferon-induced tetratricopeptide repeat (IFIT) protein family (44). Some previous studies have indicated that IFIT5 was high-expressed and negatively correlated with the prognosis in renal cell carcinoma, bladder cancer, and prostate cancer patients. IFIT5 may function as an oncogene, promote cancer invasion, metastasis, and progression by inducing epithelial–mesenchymal transition (EMT) via modulating turnover of tumor suppressive microRNAs, including miR-363, miR-99a, and miR-128 (45–48). PPARGC1A, also known as PGC-1α, functions as a master regulator of mitochondrial biogenesis and oxidative phosphorylation and plays a pivotal role in cancer cell metabolism and metastasis (49, 50). Some published studies have demonstrated that PPARGC1A was upregulated in lung cancer and invasive breast cancer, and facilitated cancer metastasis and invasion. Moreover, PPARGC1A high expression was correlated to poor prognosis in patients with lung cancer and breast cancer (49, 51). However, the opposite results have been observed in some other studies, and PPARGC1A has been suggested as a tumor suppressor that suppresses prostate cancer and melanoma cell proliferation, migration, and metastasis (52, 53). In addition, Zhang et al. observed that PPARGC1A rs2970847 C>T polymorphisms associated with HCC risk (54). Given the importance of the six RBP genes in kinds of cancer types, these genes might be potential prognostic biomarkers for patients with HCC, but detailed molecular mechanism during hepatocarcinogenesis needs further in-depth exploration.

GSEA analysis showed that some cancer-related pathways were enriched in high-risk HCC patients, such as Wnt/beta-catenin signaling, P53 pathway, PI3K-AKT-mTOR signaling, and MYC signaling. These molecular pathways have been confirmed to be implicated in HCC carcinogenesis. Autophagy can activate Wnt/β-catenin signaling and promote HCC cells metastasis and glycolysis (55). Alpha-fetoprotein (AFP) inhibited autophagy in HCC cells by activating of PI3K/Akt/mTOR signaling, thereby promoting proliferation, migration, and invasion (56). The c-Myc was a transcription factor that plays an important role in hepatocarcinogenesis, NELFE promoted HCC progression via enhancing MYC signaling (20). P53 as a tumor suppressor protein, inhibiting the p53 pathway, may promote HCC cells proliferation and inhibit apoptosis (57). 

Thanks to the great progress in microarray and next-generation sequencing technologies, a number of multigene prognostic models have been developed to predict survival for HCC patients, such as Wang et al. developed an immune-related prognostic model in HCC (58), and Li et al. developed a CIMP-associated prognostic model for HCC (59). However, RBPs-associated prognostic model for HCC has not been reported yet; this is the first study about a prognostic model in HCC patients constructed using multiple RBP genes, to our knowledge. According to our risk score signature, survival analysis displayed significant difference of OS between high- and low-risk subgroups, and usually low-risk patients had better survival than patients with high risk score. The ROC curves suggested that our prognostic model had a good accuracy, and the AUC values of 1-, 3-year were greater than 0.75 both in training and testing set. In addition, whether in the training set or testing set, TNM stage and risk score were independent prognostic indicators in HCC. Although our model has good prediction performance, there are still some limitations that need to be discussed. First, the six-RBP gene signature was built based on TCGA HCC dataset and was only validated in the ICGC HCC dataset, which has not been validated in our own clinical HCC cases cohort. Second, most HCC patients in TCGA database were Caucasian, and it is not clear whether it has the same predictive effect in non-Caucasian races. Finally, our study was retrospective and needs further validation by a larger prospective study.

In conclusion, we identified differently expressed RBP genes and constructed a promising six-RBP gene prognostic signature to predict clinical outcomes for HCC patients. This risk score signature was proven to have good predictive ability and function as an independent prognostic indicator for HCC patients, contributing to guided clinical decision making and personalized treatment. Moreover, this study would further help us understand the prognostic value and biological function of RBPs in HCC.

## Data Availability Statement

Publicly available datasets were analyzed in this study. This data can be found here: The Cancer Genome Atlas (https://portal.gdc.cancer.gov/).

## Author Contributions

YH and SC designed the study. YH, WQ, and LL carried out data extraction and statistical analysis. YH, XY, and QL drafted and modified the manuscript. All authors contributed to the article and approved the submitted version.

## Funding 

This work was supported by grants from the National Natural Science Foundation of China (2018, 81773360).

## Conflict of Interest

The authors declare that the research was conducted in the absence of any commercial or financial relationships that could be construed as a potential conflict of interest.

## Abbreviations

HCC, Hepatocellular carcinoma; RBPs, RNA binding proteins; TCGA, The Cancer Genome Atlas; ICGC, International Cancer Genome Consortium; OS, Overall survival; ROC, Receiver operating characteristic; DE RBPs, Differentially expressed RBPs; GO, Gene ontology; KEGG, Kyoto Encyclopedia of Genes and Genomes; FC, Fold change; FDR, false discovery rate; PPI, Protein-protein interaction; MCODE, Molecular Complex Detection; LASSO, Least absolute shrinkage selection operator; AUC, Area under the curve; GSEA, Gene set enrichment analysis; HPA, Human Protein Atlas; BP, Biological process; CC, Cell component; MF, Molecular function; NES, Normalized enrichment score.
