# Supplementary material for: A Novel RNA Binding Protein-Related Prognostic Signature for Hepatocellular Carcinoma
Source: Front Oncol. 2020 Oct 28;10:580513. doi: 10.3389/fonc.2020.580513 (PMC7673432; doi:10.3389/fonc.2020.580513)
Supplement: Supplementary file 1 [file Table_1.docx]

**Supplementary Table 1. The differently expressed RBPs.**

| **Gene** | | **Normal** | | **HCC** | **logFC** | **p-Value** | **FDR** |
| --- | --- | --- | --- | --- | --- | --- | --- |
| TRIM71 | 0.002794 | | 0.934468 | | 8.385784 | 5.09E-13 | 3.42E-12 |
| ZNF579 | 1.741972 | | 2.948376 | | 0.759199 | 1.58E-06 | 4.00E-06 |
| MSI1 | 0.249093 | | 2.385502 | | 3.259537 | 0.000443 | 0.000775 |
| MSI2 | 0.906208 | | 1.310707 | | 0.532431 | 3.83E-06 | 9.14E-06 |
| RBMS1 | 3.238789 | | 2.079448 | | -0.63925 | 4.66E-11 | 2.18E-10 |
| RBMS3 | 1.008284 | | 0.349373 | | -1.52906 | 9.54E-25 | 1.16E-22 |
| RRP15 | 1.714889 | | 2.451925 | | 0.5158 | 1.45E-10 | 6.39E-10 |
| L1TD1 | 0.03767 | | 0.023746 | | -0.66569 | 0.002191 | 0.003449 |
| NMD3 | 16.23933 | | 10.19607 | | -0.67148 | 9.16E-19 | 2.12E-17 |
| SRSF12 | 0.129815 | | 0.243708 | | 0.908697 | 0.000268 | 0.000491 |
| SRSF8 | 11.48658 | | 5.91018 | | -0.95868 | 1.36E-25 | 2.28E-23 |
| DAP3 | 17.34214 | | 25.10286 | | 0.53357 | 3.54E-13 | 2.45E-12 |
| TPR | 4.453371 | | 6.720498 | | 0.59367 | 2.16E-10 | 9.11E-10 |
| METTL3 | 2.134041 | | 3.259715 | | 0.611158 | 3.45E-17 | 5.39E-16 |
| AKAP8L | 4.074829 | | 6.525311 | | 0.679307 | 3.03E-16 | 3.99E-15 |
| ELAVL2 | 0.004664 | | 0.023392 | | 2.326405 | 3.10E-08 | 1.02E-07 |
| ELAVL3 | 0.00385 | | 0.01105 | | 1.521272 | 2.39E-05 | 5.06E-05 |
| DDX41 | 9.970905 | | 14.49578 | | 0.539837 | 6.30E-17 | 9.50E-16 |
| PUSL1 | 2.697744 | | 4.249384 | | 0.6555 | 1.52E-05 | 3.34E-05 |
| CLASRP | 3.845322 | | 5.752504 | | 0.581085 | 8.70E-12 | 4.58E-11 |
| TOP3B | 0.030923 | | 0.056689 | | 0.874374 | 1.91E-06 | 4.78E-06 |
| SMG9 | 1.294686 | | 2.690353 | | 1.055194 | 3.94E-21 | 1.51E-19 |
| MKRN3 | 0.009251 | | 0.124898 | | 3.754987 | 1.33E-09 | 5.13E-09 |
| DHX57 | 1.081349 | | 1.77142 | | 0.712074 | 1.99E-21 | 8.36E-20 |
| TCOF1 | 1.717359 | | 3.317792 | | 0.950032 | 7.06E-22 | 3.95E-20 |
| MRPL53 | 1.154058 | | 2.165888 | | 0.908243 | 1.00E-16 | 1.44E-15 |
| REPIN1 | 18.58852 | | 28.55858 | | 0.619513 | 6.47E-11 | 2.95E-10 |
| ZC3H12C | 1.180293 | | 0.649271 | | -0.86225 | 2.88E-17 | 4.66E-16 |
| ZC3H12A | 7.119418 | | 4.488879 | | -0.6654 | 1.81E-06 | 4.56E-06 |
| TRMT61A | 4.699728 | | 6.747598 | | 0.521797 | 7.59E-08 | 2.37E-07 |
| PABPN1L | 0.016982 | | 0.008523 | | -0.99451 | 0.000539 | 0.000932 |
| TRMU | 1.519411 | | 2.664965 | | 0.810604 | 9.06E-14 | 7.16E-13 |
| CD3EAP | 0.726176 | | 1.045722 | | 0.526107 | 2.91E-06 | 7.11E-06 |
| RDM1 | 0.042426 | | 0.464633 | | 3.453066 | 4.60E-22 | 2.94E-20 |
| XPO5 | 2.268792 | | 4.12872 | | 0.86377 | 8.39E-22 | 4.51E-20 |
| GSPT2 | 5.61926 | | 2.487854 | | -1.17548 | 3.87E-19 | 9.44E-18 |
| EIF2D | 4.746334 | | 7.139944 | | 0.589099 | 1.77E-14 | 1.60E-13 |
| DARS2 | 4.979817 | | 8.327876 | | 0.741856 | 4.00E-12 | 2.22E-11 |
| SNRPD2 | 39.97746 | | 62.48422 | | 0.644305 | 1.16E-13 | 9.06E-13 |
| DICER1 | 4.44654 | | 2.733644 | | -0.70186 | 9.53E-18 | 1.71E-16 |
| SAMD4B | 5.142533 | | 7.43689 | | 0.53222 | 1.33E-16 | 1.89E-15 |
| SAMD4A | 2.676886 | | 1.506118 | | -0.82972 | 6.19E-16 | 7.36E-15 |
| ENDOG | 11.89314 | | 7.107157 | | -0.74279 | 4.11E-10 | 1.69E-09 |
| EXOG | 0.498888 | | 0.757187 | | 0.601934 | 8.60E-11 | 3.85E-10 |
| MRPL24 | 48.44521 | | 76.30862 | | 0.655492 | 1.11E-08 | 3.88E-08 |
| CDC40 | 2.819697 | | 1.806998 | | -0.64194 | 5.11E-16 | 6.18E-15 |
| RCL1 | 22.40095 | | 7.422615 | | -1.59356 | 3.57E-22 | 2.58E-20 |
| ADAT2 | 0.396369 | | 0.611658 | | 0.625882 | 1.19E-06 | 3.08E-06 |
| BOP1 | 7.519213 | | 19.14839 | | 1.34857 | 1.77E-17 | 3.07E-16 |
| POLR1E | 12.33479 | | 6.422973 | | -0.94142 | 9.23E-22 | 4.56E-20 |
| ZC3H3 | 4.504468 | | 9.182186 | | 1.027481 | 1.28E-17 | 2.26E-16 |
| NELFE | 11.00228 | | 24.95126 | | 1.18131 | 2.15E-21 | 8.76E-20 |
| APOBEC2 | 0.04924 | | 0.146589 | | 1.573877 | 5.70E-06 | 1.33E-05 |
| PATL1 | 12.68955 | | 8.705514 | | -0.54364 | 1.86E-12 | 1.12E-11 |
| EDC3 | 2.502402 | | 3.989404 | | 0.67286 | 1.78E-20 | 5.55E-19 |
| EIF5A | 164.0923 | | 100.9902 | | -0.70029 | 8.54E-20 | 2.34E-18 |
| EIF5A2 | 0.521646 | | 1.199027 | | 1.20072 | 0.022971 | 0.03085 |
| DAZ3 | 9.69E-05 | | 6.00E-05 | | -0.69145 | 5.14E-05 | 0.000102 |
| SPATS2 | 0.877269 | | 2.408017 | | 1.456755 | 1.85E-26 | 4.97E-24 |
| MAEL | 0.074764 | | 1.139456 | | 3.929857 | 0.011105 | 0.015716 |
| TIPARP | 8.486049 | | 4.185566 | | -1.01967 | 2.02E-16 | 2.83E-15 |
| POLR2J3 | 0.148234 | | 0.211596 | | 0.513436 | 0.001384 | 0.002242 |
| ZCCHC14 | 7.743443 | | 5.358677 | | -0.5311 | 4.17E-11 | 1.96E-10 |
| ZCCHC2 | 4.516172 | | 2.524297 | | -0.83922 | 4.90E-20 | 1.46E-18 |
| LARS | 5.029743 | | 7.651092 | | 0.605181 | 3.06E-19 | 7.61E-18 |
| TSEN15 | 5.807549 | | 8.341917 | | 0.52245 | 3.31E-10 | 1.38E-09 |
| LSM4 | 18.77306 | | 31.11557 | | 0.728973 | 2.89E-14 | 2.50E-13 |
| MRPL34 | 54.56076 | | 34.81782 | | -0.64804 | 7.95E-16 | 9.13E-15 |
| SUGP2 | 1.992576 | | 3.296819 | | 0.726439 | 6.32E-15 | 6.15E-14 |
| DDX49 | 10.64614 | | 16.7625 | | 0.654908 | 2.45E-17 | 4.11E-16 |
| SFSWAP | 1.708078 | | 2.604669 | | 0.608726 | 1.49E-19 | 3.76E-18 |
| MRPS12 | 13.4134 | | 19.21719 | | 0.518723 | 3.53E-06 | 8.52E-06 |
| EXOSC4 | 15.42828 | | 23.59778 | | 0.613074 | 0.00041 | 0.000721 |
| EXOSC6 | 5.631335 | | 3.880149 | | -0.53736 | 3.51E-12 | 2.01E-11 |
| PTRH1 | 0.010589 | | 0.018473 | | 0.802792 | 0.001776 | 0.002854 |
| INTS8 | 1.54893 | | 2.897242 | | 0.903408 | 9.74E-19 | 2.22E-17 |
| CASC3 | 6.003459 | | 8.809475 | | 0.553262 | 1.86E-15 | 2.00E-14 |
| MRPL54 | 58.41599 | | 38.48701 | | -0.60199 | 9.91E-16 | 1.13E-14 |
| GTPBP3 | 2.201052 | | 3.195655 | | 0.537918 | 2.79E-13 | 2.01E-12 |
| AZGP1 | 1007.151 | | 313.722 | | -1.68272 | 4.02E-25 | 6.00E-23 |
| NSUN6 | 14.59248 | | 6.132296 | | -1.25073 | 3.54E-22 | 2.58E-20 |
| RRP9 | 7.790431 | | 11.83635 | | 0.603449 | 1.69E-12 | 1.02E-11 |
| PUS1 | 2.953304 | | 4.446919 | | 0.590476 | 3.89E-08 | 1.26E-07 |
| TDRKH | 1.022483 | | 2.187331 | | 1.097095 | 5.76E-14 | 4.74E-13 |
| RBM44 | 0.031899 | | 0.047527 | | 0.575227 | 0.002634 | 0.00409 |
| OASL | 19.32127 | | 6.980209 | | -1.46885 | 0.005959 | 0.008727 |
| OAS1 | 16.81882 | | 8.47505 | | -0.98878 | 1.16E-06 | 3.01E-06 |
| OAS2 | 15.38139 | | 5.767428 | | -1.41519 | 0.000272 | 0.000496 |
| RPP14 | 3.134449 | | 2.165964 | | -0.5332 | 4.15E-16 | 5.26E-15 |
| TRMT6 | 2.301555 | | 3.438296 | | 0.579085 | 2.05E-14 | 1.81E-13 |
| PRPF3 | 3.191032 | | 6.108348 | | 0.936759 | 5.26E-21 | 1.87E-19 |
| ZCCHC24 | 16.52765 | | 7.175553 | | -1.20372 | 1.15E-20 | 3.69E-19 |
| RPS21 | 177.9226 | | 255.3747 | | 0.521366 | 7.22E-13 | 4.75E-12 |
| MBNL2 | 29.25617 | | 10.42387 | | -1.48885 | 3.78E-18 | 7.70E-17 |
| ALYREF | 16.33137 | | 24.69843 | | 0.596774 | 1.14E-07 | 3.43E-07 |
| PUS7 | 1.674118 | | 2.518453 | | 0.589136 | 1.59E-09 | 6.06E-09 |
| NOL3 | 4.043313 | | 6.921367 | | 0.775519 | 2.58E-05 | 5.45E-05 |
| SMAD1 | 2.385064 | | 1.352514 | | -0.81838 | 4.38E-13 | 2.99E-12 |
| SMAD7 | 4.268079 | | 2.78085 | | -0.61806 | 5.10E-11 | 2.36E-10 |
| SMAD6 | 1.440246 | | 0.711153 | | -1.01808 | 6.68E-18 | 1.28E-16 |
| PRPF39 | 1.705342 | | 2.453434 | | 0.524741 | 6.32E-11 | 2.89E-10 |
| ADAD2 | 0.036286 | | 0.023841 | | -0.60597 | 1.69E-08 | 5.75E-08 |
| CLK2 | 4.268814 | | 8.393149 | | 0.975377 | 5.30E-22 | 3.09E-20 |
| IGF2BP1 | 0.013613 | | 1.583525 | | 6.862011 | 2.97E-15 | 3.10E-14 |
| IGF2BP2 | 0.802315 | | 3.540447 | | 2.14169 | 1.28E-05 | 2.85E-05 |
| IGF2BP3 | 0.017896 | | 0.413246 | | 4.529282 | 6.03E-11 | 2.77E-10 |
| UBAP2 | 1.338545 | | 1.935639 | | 0.532144 | 3.02E-11 | 1.46E-10 |
| UBAP2L | 7.476431 | | 14.04578 | | 0.909715 | 1.62E-21 | 7.02E-20 |
| SNRPE | 18.59672 | | 35.03921 | | 0.913922 | 3.52E-21 | 1.39E-19 |
| NSUN5 | 4.249602 | | 7.219787 | | 0.764628 | 3.74E-18 | 7.70E-17 |
| MTG1 | 1.302829 | | 1.946277 | | 0.579069 | 3.64E-06 | 8.77E-06 |
| RPUSD1 | 3.991941 | | 5.99555 | | 0.586802 | 1.77E-11 | 8.96E-11 |
| NHP2 | 18.34574 | | 26.75706 | | 0.544475 | 1.67E-12 | 1.02E-11 |
| SUB1 | 10.40529 | | 15.1004 | | 0.537269 | 1.57E-09 | 6.02E-09 |
| PARP1 | 13.18525 | | 19.12738 | | 0.536714 | 1.05E-08 | 3.65E-08 |
| SF3A2 | 9.192035 | | 15.87849 | | 0.788618 | 1.44E-14 | 1.33E-13 |
| AFF1 | 6.083749 | | 3.887635 | | -0.64607 | 6.85E-14 | 5.58E-13 |
| AFF4 | 12.81541 | | 7.253374 | | -0.82116 | 2.25E-16 | 3.09E-15 |
| AFF2 | 0.004923 | | 0.102896 | | 4.385592 | 0.000259 | 0.000474 |
| SNRPD1 | 3.99057 | | 6.100195 | | 0.61226 | 1.39E-09 | 5.35E-09 |
| SNRPC | 38.36232 | | 63.51906 | | 0.7275 | 5.91E-20 | 1.69E-18 |
| RBM3 | 18.51217 | | 32.21779 | | 0.799384 | 8.01E-13 | 5.20E-12 |
| CNOT7 | 4.968076 | | 3.34259 | | -0.57172 | 1.94E-14 | 1.72E-13 |
| A1CF | 41.56374 | | 18.92466 | | -1.13506 | 5.07E-21 | 1.87E-19 |
| RBM47 | 11.53877 | | 7.323648 | | -0.65586 | 2.31E-12 | 1.36E-11 |
| GPATCH4 | 4.648846 | | 7.738231 | | 0.735131 | 1.55E-14 | 1.41E-13 |
| RBM20 | 0.042181 | | 0.118309 | | 1.487915 | 0.000321 | 0.000573 |
| KHDC1 | 0.020207 | | 0.068298 | | 1.756995 | 0.018819 | 0.025737 |
| RPL10L | 0.013129 | | 0.401577 | | 4.934798 | 0.000962 | 0.001598 |
| EPRS | 11.27148 | | 17.84537 | | 0.662873 | 1.60E-13 | 1.19E-12 |
| DHX58 | 7.448387 | | 4.622221 | | -0.68834 | 4.15E-09 | 1.52E-08 |
| DDX58 | 5.363015 | | 3.510038 | | -0.61156 | 0.000102 | 0.000198 |
| IFIH1 | 6.737079 | | 4.385292 | | -0.61945 | 0.00018 | 0.000337 |
| TEX13A | 0.001086 | | 0.000688 | | -0.65758 | 2.12E-05 | 4.52E-05 |
| BARD1 | 0.29994 | | 0.617981 | | 1.042888 | 3.56E-12 | 2.02E-11 |
| RUVBL1 | 2.915984 | | 4.17159 | | 0.516615 | 1.67E-12 | 1.02E-11 |
| INTS10 | 8.633846 | | 4.570361 | | -0.9177 | 5.14E-22 | 3.09E-20 |
| RANBP6 | 4.99306 | | 3.471815 | | -0.52423 | 7.28E-14 | 5.89E-13 |
| DNMT3B | 0.234605 | | 0.630267 | | 1.425728 | 5.13E-15 | 5.03E-14 |
| RNASE9 | 0.000798 | | 0.000429 | | -0.89485 | 0.000919 | 0.001535 |
| TDRD15 | 0.100812 | | 0.060634 | | -0.73347 | 3.12E-08 | 1.02E-07 |
| WDR4 | 1.710585 | | 3.037086 | | 0.828198 | 1.46E-13 | 1.11E-12 |
| ILF2 | 30.6087 | | 55.89111 | | 0.868677 | 1.10E-21 | 5.11E-20 |
| ILF3 | 7.196368 | | 12.08804 | | 0.74824 | 4.37E-22 | 2.94E-20 |
| TCERG1 | 1.690909 | | 2.663309 | | 0.655421 | 1.52E-15 | 1.64E-14 |
| NXF3 | 0.406269 | | 0.047226 | | -3.10477 | 2.71E-27 | 1.82E-24 |
| RRS1 | 9.51783 | | 15.77529 | | 0.728962 | 6.07E-07 | 1.64E-06 |
| PRKDC | 3.808437 | | 6.321553 | | 0.73108 | 1.88E-09 | 7.12E-09 |
| FASTK | 16.21477 | | 23.04761 | | 0.507309 | 8.72E-10 | 3.46E-09 |
| ACO1 | 36.50789 | | 20.87414 | | -0.80649 | 3.53E-16 | 4.52E-15 |
| PCBP4 | 3.3355 | | 6.599402 | | 0.984432 | 2.12E-14 | 1.86E-13 |
| UNK | 2.487133 | | 3.902803 | | 0.650027 | 1.15E-15 | 1.29E-14 |
| UNKL | 0.599838 | | 1.099647 | | 0.874395 | 3.41E-10 | 1.42E-09 |
| CNBP | 217.9217 | | 127.6069 | | -0.7721 | 1.15E-19 | 3.09E-18 |
| PAIP2B | 6.151315 | | 2.247471 | | -1.45259 | 2.15E-20 | 6.56E-19 |
| DDX39A | 5.511379 | | 13.71491 | | 1.31526 | 3.65E-22 | 2.58E-20 |
| DDX39B | 3.234733 | | 6.35099 | | 0.973335 | 3.60E-11 | 1.72E-10 |
| DHX37 | 2.063603 | | 3.031858 | | 0.555037 | 7.56E-13 | 4.96E-12 |
| RNASE3 | 0.048532 | | 0.016977 | | -1.51533 | 1.97E-07 | 5.80E-07 |
| RNASE4 | 16.67677 | | 7.135402 | | -1.22477 | 8.04E-20 | 2.25E-18 |
| ANG | 725.0086 | | 304.0389 | | -1.25374 | 8.33E-18 | 1.55E-16 |
| RBMXL1 | 4.534691 | | 2.280851 | | -0.99143 | 5.37E-20 | 1.57E-18 |
| PRR3 | 1.565941 | | 2.51143 | | 0.681479 | 1.69E-12 | 1.02E-11 |
| RPL26 | 92.85726 | | 63.33168 | | -0.55209 | 1.51E-13 | 1.14E-12 |
| EIF3CL | 0.035874 | | 0.056387 | | 0.652421 | 0.003734 | 0.00561 |
| G3BP2 | 11.8782 | | 8.370821 | | -0.50488 | 2.83E-12 | 1.65E-11 |
| DHX34 | 1.934063 | | 4.06171 | | 1.070453 | 1.03E-22 | 8.61E-21 |
| SRRM3 | 0.054179 | | 0.592054 | | 3.449922 | 4.70E-06 | 1.11E-05 |
| CSDC2 | 0.371589 | | 0.158224 | | -1.23174 | 3.38E-12 | 1.94E-11 |
| RNPC3 | 0.530283 | | 0.880765 | | 0.731995 | 8.48E-09 | 3.02E-08 |
| TRIM25 | 3.119692 | | 5.253908 | | 0.751987 | 1.02E-11 | 5.35E-11 |
| DDX19B | 4.955503 | | 2.662577 | | -0.89621 | 8.91E-22 | 4.56E-20 |
| BZW2 | 5.159683 | | 7.617497 | | 0.562035 | 3.25E-07 | 9.11E-07 |
| BZW1 | 17.76043 | | 10.6906 | | -0.73232 | 3.34E-18 | 7.05E-17 |
| ZFP36 | 297.9566 | | 65.405 | | -2.18763 | 6.14E-24 | 6.34E-22 |
| ZFP36L1 | 103.9934 | | 44.97936 | | -1.20916 | 5.28E-21 | 1.87E-19 |
| ZFP36L2 | 49.67102 | | 32.72423 | | -0.60204 | 7.37E-07 | 1.97E-06 |
| DROSHA | 2.655778 | | 3.812107 | | 0.521454 | 4.26E-16 | 5.30E-15 |
| MRPL37 | 52.85448 | | 35.72327 | | -0.56516 | 6.41E-17 | 9.54E-16 |
| PUS10 | 2.145745 | | 1.21845 | | -0.81643 | 1.69E-18 | 3.72E-17 |
| TRMT11 | 5.766063 | | 3.616894 | | -0.67284 | 1.28E-12 | 8.01E-12 |
| THOC5 | 1.765478 | | 2.802878 | | 0.66685 | 8.33E-14 | 6.66E-13 |
| TSEN54 | 3.952821 | | 7.535366 | | 0.930795 | 6.41E-19 | 1.51E-17 |
| TARBP1 | 1.21305 | | 3.94544 | | 1.701547 | 1.19E-25 | 2.28E-23 |
| AGO2 | 0.930597 | | 1.752888 | | 0.913506 | 5.34E-14 | 4.45E-13 |
| AGO3 | 0.650324 | | 0.459165 | | -0.50214 | 3.28E-14 | 2.81E-13 |
| LARP4 | 13.79216 | | 8.560092 | | -0.68815 | 7.80E-17 | 1.14E-15 |
| EZH2 | 0.566551 | | 2.624728 | | 2.211891 | 1.33E-26 | 4.47E-24 |
| SNRPA | 11.77964 | | 17.29509 | | 0.554068 | 2.86E-12 | 1.66E-11 |
| HEATR1 | 1.478762 | | 2.600138 | | 0.814198 | 2.63E-13 | 1.91E-12 |
| AARSD1 | 0.66997 | | 0.998447 | | 0.575589 | 1.68E-10 | 7.22E-10 |
| INTS3 | 2.690697 | | 4.186738 | | 0.637847 | 2.13E-08 | 7.14E-08 |
| EIF2AK4 | 5.368773 | | 3.589523 | | -0.5808 | 1.47E-13 | 1.11E-12 |
| QKI | 5.03857 | | 3.338592 | | -0.59377 | 7.89E-16 | 9.13E-15 |
| IPO9 | 3.527632 | | 5.567436 | | 0.658313 | 1.55E-14 | 1.41E-13 |
| CPSF4 | 3.125154 | | 5.17926 | | 0.728819 | 4.26E-16 | 5.30E-15 |
| CSTF2 | 2.327883 | | 4.063599 | | 0.803739 | 2.46E-16 | 3.27E-15 |
| PPARGC1A | 11.65787 | | 5.270258 | | -1.14536 | 6.01E-12 | 3.24E-11 |
| ZC3H13 | 5.555709 | | 3.800321 | | -0.54785 | 4.17E-11 | 1.96E-10 |
| UPF3B | 2.192439 | | 3.906844 | | 0.833467 | 5.31E-18 | 1.05E-16 |
| RBM24 | 0.341027 | | 1.76562 | | 2.372218 | 1.66E-10 | 7.20E-10 |
| IGHMBP2 | 1.984398 | | 3.185245 | | 0.682703 | 4.32E-05 | 8.69E-05 |
| VARS | 11.33392 | | 17.38296 | | 0.617027 | 1.15E-12 | 7.23E-12 |
| INTS7 | 3.445162 | | 5.09683 | | 0.565028 | 3.82E-10 | 1.58E-09 |
| ATXN2L | 7.527403 | | 10.85215 | | 0.527757 | 7.14E-10 | 2.87E-09 |
| PPIL4 | 6.991776 | | 4.399651 | | -0.66827 | 6.47E-17 | 9.54E-16 |
| LSM11 | 0.545695 | | 0.851501 | | 0.641914 | 1.61E-09 | 6.12E-09 |
| AARS2 | 2.819 | | 4.708462 | | 0.740072 | 6.40E-19 | 1.51E-17 |
| RNF17 | 0.001944 | | 0.096479 | | 5.633446 | 0.007036 | 0.010216 |
| TDRD1 | 0.094868 | | 0.062551 | | -0.60089 | 3.22E-13 | 2.28E-12 |
| ENOX1 | 0.09859 | | 0.298648 | | 1.598941 | 0.002324 | 0.003647 |
| CMTR2 | 2.655116 | | 1.576657 | | -0.75191 | 1.09E-20 | 3.56E-19 |
| TRMT1 | 5.337424 | | 9.065085 | | 0.764177 | 9.18E-18 | 1.67E-16 |
| GPATCH1 | 1.456455 | | 2.13096 | | 0.549042 | 2.67E-13 | 1.93E-12 |
| EEF1A2 | 3.298226 | | 50.56063 | | 3.938252 | 2.06E-05 | 4.41E-05 |
| R3HDM1 | 1.493629 | | 2.21042 | | 0.565498 | 1.31E-15 | 1.45E-14 |
| METTL14 | 3.575806 | | 2.518659 | | -0.50561 | 9.98E-16 | 1.13E-14 |
| MRPL32 | 15.51316 | | 9.886996 | | -0.64989 | 7.95E-21 | 2.67E-19 |
| WDR46 | 10.18765 | | 14.90239 | | 0.548722 | 1.82E-14 | 1.64E-13 |
| CMSS1 | 2.343831 | | 3.363656 | | 0.521162 | 1.00E-09 | 3.94E-09 |
| UTP14A | 2.716878 | | 4.079404 | | 0.586409 | 2.34E-13 | 1.71E-12 |
| UTP14C | 4.40239 | | 2.875729 | | -0.61436 | 4.89E-13 | 3.32E-12 |
| TRUB2 | 11.4187 | | 7.915595 | | -0.52863 | 8.71E-15 | 8.30E-14 |
| PRIM1 | 1.465525 | | 3.350573 | | 1.192991 | 2.29E-14 | 2.00E-13 |
| CPSF1 | 8.618365 | | 13.81784 | | 0.681046 | 4.12E-12 | 2.28E-11 |
| DDX3X | 30.99143 | | 16.96291 | | -0.86949 | 6.74E-16 | 7.95E-15 |
| NOP56 | 7.163574 | | 11.72838 | | 0.711252 | 5.23E-13 | 3.49E-12 |
| MRM1 | 3.038856 | | 4.507488 | | 0.568795 | 6.67E-08 | 2.08E-07 |
| DXO | 4.080325 | | 7.221775 | | 0.823669 | 3.90E-17 | 6.01E-16 |
| NOL12 | 0.549045 | | 1.089442 | | 0.988594 | 2.75E-17 | 4.50E-16 |
| DNMT1 | 1.798338 | | 3.921409 | | 1.124708 | 4.46E-16 | 5.49E-15 |
| SNRPA1 | 3.898551 | | 5.667746 | | 0.539837 | 9.89E-08 | 3.02E-07 |
| PUF60 | 22.62758 | | 35.36184 | | 0.644111 | 2.45E-11 | 1.21E-10 |
| EIF5 | 35.75933 | | 20.21929 | | -0.82259 | 1.19E-19 | 3.12E-18 |
| RALY | 9.026893 | | 13.63934 | | 0.595472 | 3.88E-15 | 3.94E-14 |
| SEPSECS | 9.92536 | | 5.196344 | | -0.93362 | 6.52E-21 | 2.25E-19 |
| NPM1 | 55.05114 | | 82.02368 | | 0.575268 | 6.00E-10 | 2.43E-09 |
| NPM3 | 7.705219 | | 13.26819 | | 0.784063 | 1.41E-06 | 3.60E-06 |
| NPM2 | 0.623489 | | 2.061405 | | 1.725191 | 0.034606 | 0.045298 |
| NUPL2 | 2.715767 | | 3.916239 | | 0.528109 | 2.09E-17 | 3.56E-16 |
| SNRPB | 54.6293 | | 110.2936 | | 1.013602 | 9.50E-22 | 4.56E-20 |
| POLR2K | 17.42254 | | 28.32639 | | 0.701192 | 5.36E-14 | 4.45E-13 |
| MRPL13 | 7.425438 | | 10.78795 | | 0.538872 | 3.90E-05 | 7.91E-05 |
| MRPL46 | 5.164146 | | 3.203795 | | -0.68875 | 3.43E-15 | 3.55E-14 |
| TTF2 | 0.367621 | | 0.620896 | | 0.756135 | 1.44E-10 | 6.33E-10 |
| PUS3 | 13.43038 | | 8.939235 | | -0.58728 | 3.53E-15 | 3.62E-14 |
| RBM7 | 3.154385 | | 1.752641 | | -0.84783 | 9.70E-26 | 2.17E-23 |
| UTP3 | 16.10445 | | 9.952884 | | -0.69427 | 1.24E-16 | 1.77E-15 |
| WDR83 | 1.93743 | | 2.970737 | | 0.616677 | 6.93E-09 | 2.50E-08 |
| SNRNP70 | 18.69981 | | 28.96329 | | 0.631202 | 7.63E-13 | 4.97E-12 |
| MRPL39 | 22.69048 | | 13.41482 | | -0.75826 | 9.21E-24 | 8.84E-22 |
| TLR8 | 0.547701 | | 0.280196 | | -0.96695 | 2.30E-10 | 9.69E-10 |
| TLR3 | 2.549741 | | 1.092956 | | -1.22212 | 2.08E-18 | 4.51E-17 |
| BRCA1 | 0.58079 | | 0.893424 | | 0.621328 | 0.000274 | 0.0005 |
| DQX1 | 0.011822 | | 0.734614 | | 5.957475 | 2.98E-07 | 8.38E-07 |
| TDRD12 | 0.008189 | | 0.018923 | | 1.208312 | 0.006641 | 0.009674 |
| ZNF473 | 0.754955 | | 1.106918 | | 0.552086 | 2.50E-12 | 1.47E-11 |
| CELF3 | 0.001044 | | 0.020674 | | 4.308149 | 2.32E-11 | 1.15E-10 |
| DDX53 | 0.000577 | | 0.177848 | | 8.267004 | 7.07E-05 | 0.000138 |
| CPEB2 | 4.818114 | | 3.012005 | | -0.67774 | 1.41E-11 | 7.22E-11 |
| CPEB3 | 6.863387 | | 1.34056 | | -2.35608 | 6.70E-28 | 9.00E-25 |
| CPEB4 | 10.35574 | | 5.193525 | | -0.99565 | 5.98E-17 | 9.13E-16 |
| SECISBP2L | 4.784331 | | 3.207502 | | -0.57687 | 3.72E-13 | 2.56E-12 |
| EEF1E1 | 2.304838 | | 3.422421 | | 0.570352 | 1.16E-05 | 2.59E-05 |
| IARS | 5.382273 | | 7.828164 | | 0.540458 | 1.02E-08 | 3.57E-08 |
| DDX60 | 5.231739 | | 2.896605 | | -0.85293 | 0.000291 | 0.000525 |
| DDX60L | 1.667989 | | 0.960046 | | -0.79693 | 1.54E-10 | 6.71E-10 |
| MAZ | 6.594285 | | 10.68944 | | 0.696899 | 1.16E-11 | 5.98E-11 |
| PABPC3 | 0.054367 | | 0.112807 | | 1.053057 | 3.68E-05 | 7.50E-05 |
| PABPC1L | 1.42076 | | 4.403291 | | 1.63192 | 2.28E-16 | 3.09E-15 |
| PABPC1L2B | 0.001429 | | 0.000975 | | -0.55077 | 0.000629 | 0.001073 |
| PABPC4L | 0.038553 | | 0.102017 | | 1.403887 | 3.97E-12 | 2.21E-11 |
| PABPC1 | 114.2706 | | 181.9073 | | 0.67075 | 1.30E-06 | 3.34E-06 |
| MEX3A | 0.339655 | | 1.060625 | | 1.642773 | 6.32E-12 | 3.38E-11 |
| MEX3B | 0.210899 | | 0.31625 | | 0.584514 | 0.013186 | 0.018446 |
| MRPS23 | 3.382594 | | 5.492389 | | 0.699304 | 1.66E-18 | 3.71E-17 |
| RPL39L | 1.98969 | | 5.664884 | | 1.509503 | 0.006677 | 0.009715 |
| RPSA | 62.48538 | | 91.82309 | | 0.555338 | 3.54E-11 | 1.70E-10 |
| SMG5 | 11.24141 | | 26.0227 | | 1.210948 | 1.44E-21 | 6.45E-20 |
| NR0B1 | 0.002828 | | 0.339196 | | 6.906218 | 0.01289 | 0.018052 |
| FUS | 9.631624 | | 14.29136 | | 0.569292 | 5.68E-12 | 3.08E-11 |
| FBL | 29.47467 | | 42.08499 | | 0.51383 | 3.56E-07 | 9.88E-07 |
| NOP14 | 15.45948 | | 10.65134 | | -0.53746 | 6.59E-18 | 1.28E-16 |
| SNIP1 | 3.748377 | | 2.567005 | | -0.54618 | 1.11E-14 | 1.04E-13 |
| SRSF5 | 33.84051 | | 22.23243 | | -0.60609 | 3.26E-17 | 5.21E-16 |
| TERT | 0.002769 | | 1.380221 | | 8.961399 | 1.10E-24 | 1.23E-22 |
| DCAF13 | 2.001952 | | 3.838277 | | 0.939052 | 5.09E-13 | 3.42E-12 |
| POP1 | 0.724426 | | 1.060107 | | 0.549299 | 1.57E-09 | 6.02E-09 |
| CDK9 | 27.36043 | | 16.49215 | | -0.73031 | 3.36E-18 | 7.05E-17 |
| XRN1 | 3.383116 | | 2.270434 | | -0.57538 | 1.40E-13 | 1.07E-12 |
| RRP12 | 2.344244 | | 3.547885 | | 0.597836 | 7.96E-08 | 2.46E-07 |
| MRPS2 | 28.6675 | | 19.04571 | | -0.58995 | 8.75E-18 | 1.61E-16 |
| RPL22L1 | 7.440337 | | 16.27488 | | 1.129207 | 2.44E-05 | 5.16E-05 |
| RNASEH2A | 2.802313 | | 9.420481 | | 1.749183 | 1.45E-23 | 1.30E-21 |
| RBM12B | 0.683478 | | 0.975482 | | 0.513221 | 1.45E-08 | 4.98E-08 |
| ESRP2 | 15.54267 | | 8.861568 | | -0.8106 | 2.46E-16 | 3.27E-15 |
| RPP40 | 2.456079 | | 3.672065 | | 0.580235 | 4.34E-06 | 1.03E-05 |
| CNOT6 | 1.701805 | | 2.447832 | | 0.524439 | 2.02E-08 | 6.81E-08 |
| CNOT6L | 3.743903 | | 2.600428 | | -0.52579 | 3.35E-13 | 2.35E-12 |
| NAF1 | 1.321986 | | 0.901468 | | -0.55236 | 1.40E-15 | 1.53E-14 |
| LRRFIP1 | 8.750275 | | 5.619481 | | -0.63889 | 1.34E-14 | 1.25E-13 |
| EIF4E3 | 1.985567 | | 0.884968 | | -1.16585 | 2.50E-17 | 4.15E-16 |
| CTU1 | 1.138109 | | 1.715115 | | 0.591667 | 0.000191 | 0.000354 |
| GTPBP2 | 3.890524 | | 7.35751 | | 0.919253 | 2.13E-16 | 2.95E-15 |
| DDX21 | 14.72747 | | 9.747674 | | -0.59538 | 9.41E-06 | 2.14E-05 |
| RPL36A | 7.383876 | | 12.13255 | | 0.716433 | 0.000133 | 0.000253 |
| INTS6 | 1.428406 | | 0.866669 | | -0.72085 | 6.43E-15 | 6.22E-14 |
| MRPS21 | 27.43659 | | 44.58108 | | 0.70033 | 1.20E-13 | 9.28E-13 |
| SETD7 | 8.62039 | | 5.791581 | | -0.5738 | 8.49E-11 | 3.83E-10 |
| EXO1 | 0.102819 | | 1.147849 | | 3.480752 | 1.20E-26 | 4.47E-24 |
| NANOS1 | 0.068136 | | 0.402343 | | 2.561938 | 1.47E-12 | 9.08E-12 |
| NANOS3 | 0.043658 | | 0.104449 | | 1.258501 | 0.031949 | 0.042231 |
| LSM2 | 15.58449 | | 24.22572 | | 0.636429 | 2.23E-11 | 1.11E-10 |
| RPP21 | 2.390302 | | 4.220231 | | 0.820129 | 5.44E-12 | 2.96E-11 |
| IFIT1 | 27.67963 | | 10.31328 | | -1.42432 | 1.43E-07 | 4.30E-07 |
| IFIT2 | 12.63184 | | 6.376647 | | -0.98619 | 0.002414 | 0.003766 |
| IFIT5 | 6.241425 | | 4.387907 | | -0.50834 | 8.56E-10 | 3.40E-09 |
| IFIT3 | 24.02398 | | 11.61307 | | -1.04873 | 0.015127 | 0.021009 |
| PTCD1 | 0.91262 | | 1.454112 | | 0.672052 | 4.64E-10 | 1.90E-09 |
| RPL8 | 368.5442 | | 599.7074 | | 0.702421 | 1.80E-13 | 1.33E-12 |
| IMP3 | 24.3814 | | 16.05359 | | -0.60288 | 7.78E-18 | 1.47E-16 |
| LIN28B | 0.000276 | | 0.446366 | | 10.6595 | 0.000445 | 0.000777 |
| R3HCC1 | 13.92805 | | 9.019784 | | -0.62683 | 5.46E-16 | 6.55E-15 |
| MARS | 6.635815 | | 9.692416 | | 0.546583 | 1.11E-12 | 7.04E-12 |
| SF3B4 | 18.2047 | | 35.06922 | | 0.945894 | 1.39E-19 | 3.60E-18 |
| LARP1 | 13.15534 | | 18.84766 | | 0.518737 | 9.69E-10 | 3.83E-09 |
| LARP1B | 6.418256 | | 2.847932 | | -1.17227 | 7.63E-25 | 1.02E-22 |
| BAZ2B | 0.988198 | | 0.64833 | | -0.60807 | 1.92E-13 | 1.41E-12 |
| TST | 308.4544 | | 194.2319 | | -0.66728 | 3.31E-12 | 1.91E-11 |
| SETX | 5.126696 | | 3.549445 | | -0.53044 | 2.17E-11 | 1.09E-10 |
